# Oxidative and endoplasmic reticulum stress in respiratory disease

**DOI:** 10.1002/cti2.1019

**Published:** 2018-06-13

**Authors:** Alice C‐H Chen, Lucy Burr, Michael A McGuckin

**Affiliations:** ^1^ Diamantina Institute Faculty of Medicine The University of Queensland Brisbane QLD Australia; ^2^ Department of Cell and Molecular Therapy Royal Prince Alfred Hospital Sydney NSW Australia; ^3^ Department of Respiratory Medicine Mater Adult Hospital and Mater Research Institute – The University of Queensland Raymond Tce, South Brisbane QLD Australia; ^4^ Inflammatory Disease Biology and Therapeutics Group Translational Research Institute Mater Research Institute ‐ The University of Queensland Brisbane QLD Australia

**Keywords:** cystic fibrosis, endoplasmic reticulum stress, inflammation, lung disease, oxidative stress, protein misfolding, respiratory epithelium

## Abstract

Oxidative stress and endoplasmic reticulum (ER) stress are related states that can occur in cells as part of normal physiology but occur frequently in diseases involving inflammation. In this article, we review recent findings relating to the role of oxidative and ER stress in the pathophysiology of acute and chronic nonmalignant diseases of the lung, including infections, cystic fibrosis, idiopathic pulmonary fibrosis and asthma. We also explore the potential of drugs targeting oxidative and ER stress pathways to alleviate disease.

## Introduction to oxidative and ER stress

Oxidative stress and endoplasmic reticulum (ER) stress are related states that can occur in cells as part of normal physiology but which have been linked in the pathophysiology of many diseases, particularly diseases involving acute or chronic inflammation.^1^ Oxidative stress occurs when there is an imbalance between the production and degradation of reactive oxygen or nitrogen species (hereto ROS and RNS, respectively) within a cell, or when there is an excess of environmental ROS/RNS that can diffuse into the cell. One consequence of oxidative stress is disruption of the correct oxidative environment within the ER where proteins in the secretory pathway are produced, leading to misfolding of these proteins and ER stress. Interestingly, other triggers of protein misfolding in the ER result in excessive ROS production and therefore oxidative stress, further linking these states. Oxidative stress and ER stress are entwined with inflammation because inflammatory factors drive the production of ROS/RNS and because the stress activates inflammatory signalling in the affected cells, potentially setting up a forward‐feeding loop of stress and inflammation.^1^ In this article, we review recent findings relating to the role of oxidative and ER stress in the pathophysiology of acute and chronic nonmalignant diseases of the lung, and the potential of drugs targeting these pathways to alleviate disease. Although there is evidence for basal and therapy‐induced ER stress in lung cancer, and ER stress is being explored as a therapeutic target in this and other cancers,^2^ this topic will not be dealt with in this review.

### ER function and protein misfolding/ER stress

The endoplasmic reticulum (ER) is responsible for the initial steps of biosynthesis of secretory pathway proteins including folding, N‐glycosylation, disulphide bond formation, other posttranslational modifications and for protein quality control, which ensures newly made secretory pathway proteins are suitable for function on the cell surface or secretion. The ER is also important for calcium homeostasis, being the major reservoir of intracellular Ca^2+^. A range of chaperones and enzymes resident within the ER are essential for correct protein folding and maintenance of the ER biosynthetic machinery.^3^ Typically, these chaperones disengage from proteins once the correct conformation is achieved allowing exit of the protein from the ER. Although some protein misfolding occurs in all cells and increases with increasing protein complexity, when an excess of unfolded protein is present, or when intracellular Ca^2+^ levels are disturbed, the unfolded protein response (UPR) is triggered. The UPR involves a series of signalling and transcriptional events to restore ER homeostasis via decreased translation, upregulation of ER chaperones and other molecules associated with productive folding (ER‐associated folding or ERAF), and increased degradation of misfolded proteins (ER‐associated degradation or ERAD), and this has been extensively reviewed.^3^, ^4^, ^5^ Briefly, there are three major arms of the UPR regulated by (1) the ER resident protein kinase RNA‐like ER kinase (PERK) which suppresses translation via eIF2α and includes transcriptional responses via activating transcription factor‐4 (ATF4) and CCAAT/enhancer‐binding protein homologous protein (CHOP), which can lead to ER stress‐induced apoptosis; (2) the endoribonucleases inositol requiring enzyme 1 (IRE1α) (ubiquitously expressed) and IRE1β (confined to mucosal secretory cells including in the lung), which splice the X‐box‐binding protein (XBP1) mRNA leading to translation of the sXBP1 transcription factor that drives expression of chaperones and other ER resident proteins required for folding and ERAD; and (3) the transcription factor ATF6, which also promotes production of proteins enhancing ER function.

Many factors can contribute to increased rates of misfolding in the ER, including increased rates of protein synthesis, missense polymorphisms in individual proteins, alterations in the oxidative environment, energy depletion, osmotic stress, viral infection and increased temperature. Prolonged ER stress can eventually lead to inflammatory signalling and premature apoptosis through several different mechanisms.^6^ The UPR has been linked to the pathogenesis of diabetes, inflammatory bowel disease, Alzheimer's disease, Parkinson's disease and many respiratory conditions including cystic fibrosis (CF),^7^, ^8^, ^9^, ^10^ chronic obstructive pulmonary disease (COPD),^11^ asthma,^12^ idiopathic pulmonary fibrosis (IPF)^13^, ^14^ and infection.^15^, ^16^, ^17^

### Oxidative stress and redox balance

An endogenous factor that has been linked to ER stress and the UPR is the excess production of ROS and RNS disturbing the redox balance of the cell. ROS/RNS play a critical role in many cellular processes and can be produced in multiple organelles, including mitochondria, peroxisomes and ER. Mitochondria and peroxisomes contain enzymes involved in the production of ROS/RNS which in turn can be important for oxidation of other molecules and for metabolism and signal transduction. However, increased production of mitochondrial and peroxisomal ROS/RNS leads to cellular oxidative stress and can induce protein misfolding and ER stress. In a stressed ER, protein misfolding and particularly dysregulated disulphide bond formation and reduction may result in ROS accumulation, diffusion into the cytoplasm and thus cause cellular oxidative stress. Thus, ER stress and oxidative stress are intrinsically linked with each process triggering the other in differing scenarios encountered by the cell. Oxidative stress can lead to activation on inflammatory signalling pathways, including via activation of (1) the NFκB transcription factor that regulates inflammatory genes but also can regulate ROS/RNS production and degradation, and (2) the NRF2 transcription factor that primarily regulates the oxidative state of the cell but can also lead to inflammatory signalling.^18^, ^19^ Interpretation of studies of ROS/RNS on inflammatory activation pathways is complex as the large number of potential pathways affected by ROS/RNS means that both direct and indirect activation mechanisms occur. However, generally the oxidative stress‐mediated activation of these transcription factors can initiate the release of cytokines and chemokines, which further contribute to cellular dysfunction, local tissue damage and inflammatory pathologies in the airways.

### Susceptibility to stress of cell types found in the lung

The lung is constituted by many different types of cells. Because susceptibility to oxidative and ER stress varies with cell type and their differentiation/activation status, it is important to understand how each of these cell types may be affected in disease states.^20^ Therefore, we now will discuss the roles of airway epithelial cells, stromal cells and immune cells in the context of oxidative and ER stress.

#### Airway epithelial cells

Airway epithelium acts as the front‐line defence for air‐borne particulate matter and pathogens to protect the lung from foreign body damage and infection. In the healthy airway, there is a thin coating of mucus that is continuously removed from the lung via the action of cilia, and if it accumulates by cough. This airway mucus is composed mainly of mucin glycoproteins and water, and provides a matrix for a wide variety of antimicrobial molecules including antibodies, defensins, protegrins, collectins, cathelicidins, lysozyme, histatins and nitric oxide^21^, ^22^, ^23^. When the mucus barrier has increased viscosity making it resistant to mucociliary clearance, or when the barrier is deficient or depleted, the risk of opportunistic microbial infection is increased.^24^ Mucins, primarily MUC5B, are tonically secreted by the nonciliated airway epithelial surface cells, known as club cells in the small airway, and can be released in large amounts in response to inhaled material by submucosal gland mucous cells, which contain large numbers of mucin granules ready for stimulated secretion.^22^

Under appropriate stimulation, the club cells transdifferentiate and both store and hyper‐secrete mucins, typically markedly upregulating production of the MUC5AC mucin. Mucin upregulation is controlled by the transcription factor SPDEF which also upregulates other proteins required for mucin biosynthesis, expands the size and biosynthetic capacity of the ER and modulates responses to TLR ligands.^25^, ^26^ Increased production of airway mucins by mucosal epithelial cells can be stimulated by adherence of probiotic bacteria and microbial products to assist clearance of pathogens. Expression and production of mucins can also be upregulated by inflammatory cytokines including IL‐1β,^27^ IL‐4,^28^ IL‐6,^29^ IL‐9,^30^ IL‐13,^31^ TNF‐α,^32^ nitric oxide,^33^ neutrophil elastase^34^ and other uncharacterised inflammatory factors, which might contribute to pathogenesis in human inflammatory airway disorders. IRE1β can also regulate mucin production, linking the UPR to mucin expression.^35^ However, if the increased production of mucin proteins is not resolved, mucus accumulation may contribute to the pathogenesis of human inflammatory airway disorders. Overproduction of airway mucus is a common problem in many airway diseases including bronchiectasis, CF and COPD where increased concentration of mucins in mucus is a key characteristic.^36^, ^37^

The secreted mucin glycoproteins are large in size, contain highly folded cysteine‐rich domains containing multiple intra‐ and intermolecular disulphide bonds formed in the ER and therefore present a substantial challenge for correct folding in the ER.^22^ ER stress occurs when proteins misfold during biosynthesis, and ER stress in mucin‐producing cells (known as mucous cells or goblet cells depending on the tissue) can occur during inflammation and could thus be a feature of airway diseases, especially those involving mucus hypersecretion. ER stress in mucin‐producing goblet cells has been demonstrated in a mouse model, in which aberrant mucin biosynthesis due to a protein misfolding mutation in the MUC2 mucin cells leads to goblet cell ER stress and an inflammatory bowel disease‐like phenotype.^38^, ^39^ Mediators that can induce ER stress in the gut and airway epithelial cells include oxidative stress and pro‐inflammatory cytokines. It is logical to suspect that the large demand of mucin glycoprotein production and the inflammatory environment in many mucopurulent lung diseases could result in ER stress in the airway epithelial cells, which could further lead to inflammatory signalling and chronic nonresolving inflammation. We will explore the evidence for this contention in a range of respiratory conditions in this review. In addition to the mucus‐producing cells, other epithelial cells such as the ciliated cells could be subjected to oxidative and ER stress and play an important role in the pathology of airway disease.

#### Stromal cells

The lung mucosa also contains stromal cells of a mesenchymal derivation that may also be exposed to oxidative and ER stress including fibroblasts, myofibroblasts and endothelial cells. We will also explore how these cells types, which are critical determinants of the fibrosis that develops in chronic lung diseases, are affected by oxidative and ER stress in acute and chronic lung disease.

#### Immune cells

In health, multiple populations of immune cells occupy the normal respiratory mucosa and the airways themselves, and additionally, in acute and chronic respiratory disease, there can be substantial recruitment and activation of leucocytes into the respiratory mucosa. Normal resident cells are dominated by macrophages which in the healthy lung are dominated by the alveolar macrophages. Whilst alveolar macrophages have an important role in ‘house‐keeping’ in the terminal airways, they can also become an arm of innate immunity following infection. Other important leucocytes in the respiratory mucosa include dendritic cells, both regulatory and effector T cells, innate lymphoid cells and NK cells. Conspicuous recruitment of neutrophils is also typical of many chronic inflammatory conditions in the lung. Many of these cell types produce effectors, such as inflammatory cytokines, that can induce oxidative and ER stress, or directly produce ROS/RNS into the respiratory microenvironment, as is characteristic of neutrophils and activated macrophages. Each of these immune cell types is also a potential target of ER and oxidative stress that needs to be considered in the context of assessing ER and oxidative stress in respiratory disease.

## Oxidative and ER stress in infectious respiratory disease

Oxidative stress and ER stress have been reported to occur in many respiratory infections and could arise via direct effects of the pathogen on infected cells or as a consequence of the immune response to the pathogen. Because viruses hijack the secretory pathway to manufacture viral glycoproteins, they place demands on the ER which often result in substantial misfolding, ER stress and subsequent activation of the UPR. In fact, viral infection has probably shaped the evolution of the UPR such that both the UPR‐induced PERK‐mediated translational block and apoptosis can be viewed as appropriate responses to minimise viral replication. The best evidence that the UPR is important in limiting viral replication is the fact that many viruses have developed specific mechanisms to interfere with the UPR or to subvert or circumvent its mechanisms of action in order that viral replication can continue in the face of intrinsic misfolding (reviewed by Frabutt and Zheng^40^, Li *et al*.^41^, Verchot^42^). In somewhat of a contradiction, for some viruses, the action of at least some arms of the UPR actually promotes greater viral replication. The influenza A virus (IAV) is an interesting example of this phenomenon. Infection of respiratory epithelial cells with IAV activates the UPR in a distinct way with suppression of the PERK and ATF6 arms of the UPR concomitant with strong activation of IRE1α.^43^ The mechanism by which this is achieved, given the common activation mechanisms for these three major arms of the UPR, is not clear, but it is functionally important because boosted activation of IRE1α is necessary for continued viral replication.^43^ Respiratory syncytial virus (RSV) is another respiratory virus that causes ER stress (for a detailed review, see Cervantes‐Ortiz *et al*.^44^) but results in a noncanonical activation of the UPR, with in this case activation of IRE1α and ATF6, but not PERK.^45^ However, in complete contrast to IAV, in RSV infection, IRE1α suppresses viral replication.^45^

**Figure 1:**
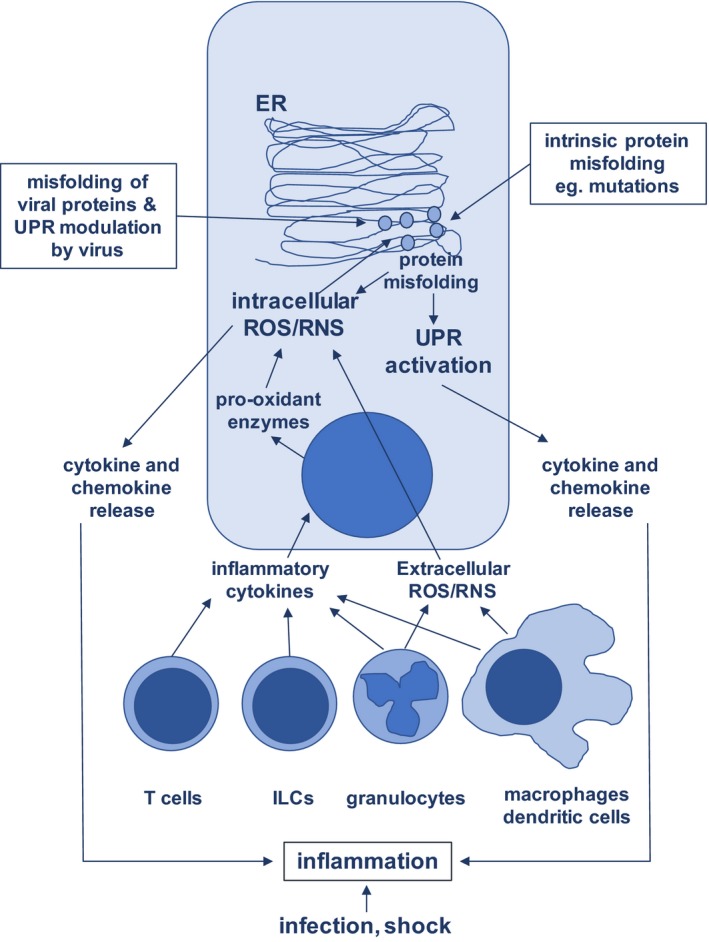
Interaction of cell intrinsic and environmental factors on the development of oxidative and ER stress in respiratory epithelial cells. ER, endoplasmic reticulum; ILC, innate lymphoid cell; ROS/RNS, reactive oxygen species, reactive nitrogen species; UPR, unfolded protein response.

Another mechanism by which infection leads to respiratory oxidative and ER stress is via mediators of the activated immune system, such as cytokines and direct production of ROS/RNS, which are discussed below in the context of chronic inflammatory lung diseases. In this context during the infectious exacerbations that frequently occur in chronic lung diseases, it is important to consider the combined effect on the ER stress pathway of infections and pre‐existing ‘noninfectious’ inflammation. As an example of experimental evidence of this concept, concomitant RSV infection exacerbates the ER stress that occurs in the murine bleomycin‐induced model of pulmonary fibrosis.^16^ Figure 1 summarises the cell intrinsic and environmental factors that potentially drive both oxidative stress and ER stress in respiratory epithelial cells.

## Oxidative and ER stress in chronic inflammatory and mucopurulent diseases

**Figure 2:**
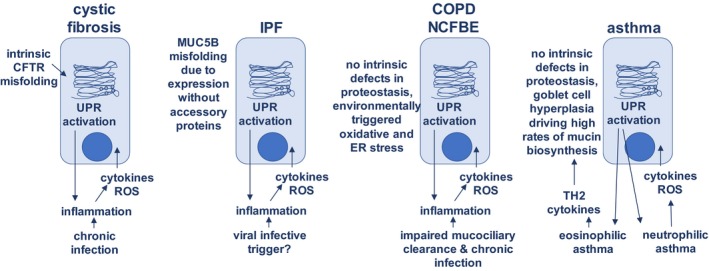
Interaction of cell intrinsic and environmental factors on the development of oxidative and ER stress in specific respiratory conditions. CFTR, cystic fibrosis conductance receptor; COPD, chronic obstructive pulmonary disease; NCFBE, noncystic fibrosis bronchiectasis; ER, endoplasmic reticulum; ROS, reactive oxygen species; TH2, T‐helper 2 immune response; UPR, unfolded protein response.

Figure 2 summarises the influences of cell intrinsic and environmental factors that may contribute to oxidative stress and ER stress in a range of respiratory diseases, and each is dealt with in the following sections.

### Cystic fibrosis (CF) and non‐CF bronchiectasis (NCFBE)

Cystic fibrosis is a disease where on face value one would predict the development of ER stress because of (1) intrinsic misfolding of the CFTR protein in the case of many mutations, including the common F508del mutation that results in misfolding of all the protein and complete absence of CFTR on the cell surface; (CFTR is normally expressed by a variety of different epithelial cell types but most highly in submucosal gland epithelium); (2) chronic bacterial infection and frequent exacerbations due to viral infection; (3) chronic complex inflammation with activation of both innate immunity and adaptive immunity; and (4) chronic mucus overproduction increasing the ER biosynthetic load of mucin‐secreting cells. Much of the work on ER stress in CF has been conducted by the Ribeiro group at University of North Carolina, and they have recently reviewed ER stress and UPR activation in CF.^46^ The most instructive data on the contribution of CFTR mutations to ER stress emanate from studies of cultured human bronchial epithelial cells (HBECs) derived from CF patients and healthy donors, separating the cells with the intrinsic defect from the inflamed and infected *in vivo* environment in the disease. Somewhat surprisingly, these studies indicate that the defects do not intrinsically drive increased ER stress or UPR activation. Whilst ER expansion and increased ER Ca^2+^ storage and signalling can be demonstrated in CF epithelial cells in short‐term cultures, these revert to normal in long‐term, nonstimulated cultures.^9^, ^47^ However, in another study, overexpression of F508del‐CFTR was reported to increase XBP1 splicing, suggesting that the mutant protein can increase ER stress.^7^ In considering the influence of the mutated protein, it is important to take into account that a large proportion (~60–80%) of wild‐type CFTR misfolds during biosynthesis due to the protein's complex transmembrane and nucleotide‐binding domains^48^; consequently, there will be a background of compensatory UPR activation in all CFTR‐expressing cells. The key question is whether the increased misfolding in the case of mutant CFTR substantially disturbs the state of proteostasis normally achieved in these respiratory epithelial cells.

Despite the lack of evidence for an intrinsic CFTR mutation driver of ER stress, there is evidence for ER stress and UPR activation in CF epithelium *in vivo*, and mucopurulent secretions from CF patients stimulate ER stress in healthy cultured HBECs with consequent XBP1‐dependent expansion of the ER^8^. Consistent with these observations, *P. aeruginosa* infection in mice induced lung inflammation and UPR activation as measured by splicing of XBP‐1 measured *in vivo* using the ERAI ER stress‐reporter transgene.^49^ Development of ER stress is unsurprising as CF lungs are chronically infected and the mucosa and lung secretions contain a broad array of inflammatory cytokines^50^, ^51^ many of which are known to drive oxidative stress and therefore ER stress (reviewed in Hasnain *et al*.^1^, ^52^).

Additionally, CF airways are characterised by pronounced neutrophil and/or macrophage accumulation and activation of these cells during infection and inflammation often results in release of ROS and RNS into the microenvironment in an attempt to control infection, increasing oxidative stress. Production of ROS is a feature of CF that has been linked with damage to the epithelium and progressive bronchiectasis and failure of lung function.^53^ Even in children with CF, there is a large amount of myeloperoxidase (MPO) produced by neutrophils and macrophages. MPO converts H_2_O_2_ into several damaging oxidants and MPO activity appears to be accompanied by depletion in the lung of counteracting reducing agent, glutathione, further enhancing the level of oxidative stress.^54^, ^55^ Activated neutrophils produce the superoxide ion (O_2_^−^) as a result of activation of the NOX2 NADPH oxidase complex, whereas airway epithelial cells primarily utilise DUOX1/2 to produce ROS (reviewed in Pongnimitprasert *et al*.^56^, Lee and Yang^57^). In contrast, inducible nitric oxide synthase (NOS2) is reduced in CF airway epithelial cells most likely in direct response to the CFTR deficiency,^58^ and there is no significant difference in the natural NOS inhibitor asymmetric dimethylarginine (ADMA) in the breath condensate from children with CF,^59^ suggesting much of the oxidative stress is driven by ROS rather than RNS. How much the altered ROS/RNS environment in the lung also affects ER stress remains unclear; however, there is evidence that the neutrophils themselves experience ER stress in CF.^60^

An interesting question in CF is whether the new class of drugs designed to facilitate folding/biogenesis of CFTR, such as Lumacaftor, influence stress in the ER.^61^, ^62^ Lumacaftor is a chaperone type drug that is designed to aid folding in the ER to enhance release of mutant CFTR to the cell surface where it has partial ion channel function. Lumacaftor is typically used in combination therapy with Ivacaftor which potentiates ion channel function of CFTR. These drugs are having a significant impact in the clinic, and although the primary benefit will be via partial restoration of the appropriate ionic environment and hydration of the airway, some benefit may be derived from reducing ER stress in highly CFTR‐expressing cells in the airway.^63^ Alternatively, it is possible that disruption of normal ER function/quality control with these modulatory drugs increases ER stress and UPR signalling, working against the desired function of the drugs. However, there are no data exploring these possibilities.

NCFBE is a chronic mucopurulent lung disease which can arise via primary ciliary dyskinesia or as a consequence of severe lung damage from infections. Although arising in the absence of any defects in CFTR, NCFBE shares many features with CF including impaired mucociliary clearance, chronic bacterial infection, frequent viral exacerbations, chronic inflammation (typically involving accumulation of activated neutrophils) and progressively declining lung function.^64^, ^65^, ^66^, ^67^ Despite these common features, our studies have failed to demonstrate any clear evidence of ER stress and UPR activation in NCFBE.

### Idiopathic pulmonary fibrosis (IPF)

Idiopathic pulmonary fibrosis is a progressive interstitial lung disease arising around the distal airway/alveoli that involves epithelial cell death, inflammation and fibrosis, with few treatment options and a high mortality rate.^68^ Although the aetiology is unclear, there are several strong genetic links to predisposition to IPF, including rare alleles in surfactant (*SFTPC*,* SFTPA2*) and telomerase pathway (*TERT*,* TERC*,* PARN* and *RTEL*) genes, and more common alleles of *MUC5B* strongly associated with a less aggressive form of IPF (reviewed by Evans *et al*.^68^). ER stress has been linked with disease development by multiple lines of evidence in human IPF and in the bleomycin‐induced murine model of IPF (for recent reviews of ER stress and IPF see Zhang *et al*.^69^ and Tanjore *et al*.^70^). Evidence for ER stress in human respiratory epithelial cells, particularly in type II pneumocytes, includes high expression of CHOP,^71^ activation of ATF6, XBP1 and ATF4.^14^ Activation of the UPR has been found in both inherited and sporadic IPF and associated with viral infection.^13^ Additionally, fibroblasts from the lungs of IPF patients show increased ER stress in response to TGFβ.^72^ IPF‐linked mutations in *SFTPC* and *SFTPA2* cause misfolding of the encoded surfactant proteins and ER stress in type II pneumocytes, providing a direct mechanistic driver for the ER stress in these forms of IPF.^13^, ^73^ Interestingly, there is substantial evidence for the importance of environmental triggers, and mice transgenic for the Δexon‐4 *SFTPC* mutation develop spontaneous lung disease,^74^ whereas those expressing L188Q *SFTPC* mutations only develop disease when exposed to low dose bleomycin.^75^ Cultured type II pneumocytes and transfected cells carrying the Δexon‐4 *SFTPC* mutation show accumulation of the SFTPC precursor protein, UPR activation and increased cell death when infected with RSV, suggesting viral infection could be a trigger for development of IPF in susceptible individuals.^76^

The common variant rs35705950 in the promoter region of *MUC5B* is carried by 9% of the European population and is the strongest risk factor for developing IPF accounting for 30–35% of the risk and also predicting asymptomatic mild fibrosis.^68^, ^77^, ^78^, ^79^ There are multiple potential explanations for how this polymorphism could lead to fibrosis (for a discussion, Evans *et al*.^68^), one of which is the ER stress pathway. Inappropriate expression of MUC5B in type II pneumocytes, particularly in the honeycomb cysts characteristic of this disease, is a feature of IPF,^80^, ^81^ and the *MUC5B* promoter polymorphism leads to enhanced *MUC5B* mRNA expression.^77^ Interestingly, this expression, potentially driven by altered transcription factor binding sites in the promoter, occurs in the absence of expression of SPDEF,^80^ which is a transcription factor that drives expression of mucin genes and a network of other genes involved in mucin biosynthesis and secretion.^82^ Taking these things together, it is inviting to propose that high expression of a complex mucin protein in a cell type lacking the appropriate ER machinery to fold and process the mucin appropriately predisposes the cell to ER stress. Perhaps this level of stress can be managed in the absence of other stressors but with ageing and accumulated environmental insults, including from viral infection, ER stress and inflammation emerge and progress with consequent fibrosis. Whilst the mucin is normally expressed only in differentiated cells, the polymorphism may drive inappropriate MUC5B expression in stem cells, as suggested by Evans *et al*.,^68^ and could lead to altered survival or function of the stem cells that are needed to appropriately renew epithelium in the terminal airways.

Animal models for IPF are imperfect, with the most commonly used being a model where fibrosis is induced by installation of bleomycin in the airway. ER stress develops in airway cells in this model and is exacerbated by viral infection, and the UPR has been associated with the differentiation status of macrophages, and intrinsic ER stress within macrophages also occurs.^16^, ^71^, ^75^, ^83^, ^84^, ^85^, ^86^

### Asthma

Asthma is a condition involving airway hyper‐responsiveness and there is emerging evidence that both oxidative stress and ER stress are features of asthma, most prominently in the neutrophil‐dominated glucocorticoid resistant severe endotype (for a review, see Kim *et al*.^87^). Genetic predisposition has also linked asthma and ER stress *via* the *ORMLD3* gene which encodes a protein that modulates function of the sarcoendoplasmic reticulum Ca^2+^ ATPase pump (SERCA) that regulates ER vs cytosolic Ca^2+^ concentrations, and thereby modulates protein folding and Ca^2+^‐signalling.^12^, ^88^, ^89^ Studies of ER stress in the human disease are rather limited but there is evidence of ER stress in both cells sampled by bronchiolar lavage and in peripheral blood leucocytes.^90^ A range of animal models of allergy and asthma have also implicated ER stress in the pathophysiology of these conditions.^90^, ^91^, ^92^, ^93^, ^94^, ^95^, ^96^ Airway goblet cell hyperplasia and mucus hypersecretion are characteristics of most forms of asthma and the increased biosynthetic load in these cells may be principal drivers of ER stress, as may the ROS/RNS released by phagocytes, particularly in neutrophilic disease. Furthermore, viral infection is a trigger for exacerbating asthma and has been shown to exacerbate ER stress in animal models of allergic asthma.^91^

### Chronic obstructive pulmonary disease (COPD) and chronic bronchitis

Chronic obstructive pulmonary disease and chronic bronchitis are characterised by inflammation, oxidative stress and mucus hypersecretion, all of which, based on earlier discussion, could drive ER stress in these conditions. There is evidence for ER stress in COPD but it is based on a limited number of validated studies.^97^, ^98^, ^99^ However, there is reasonably clear evidence that exposure of bronchial epithelial cells to cigarette smoke (the environmental driver of COPD) increases ER stress.^100^, ^101^

### α‐1‐antitrypsin (AAT) deficiency

α‐1‐Antitrypsin is primarily caused by a variety of mutations in AAT that produce disease of varying severity including liver disease and a COPD‐like emphysema that is more prevalent and severe in smokers.^102^ The liver disease arises as a result of polymerisation of AAT in hepatocytes, and accumulation within the ER and subsequent ER stress, with the hepatocyte stress driving chronic hepatitis potentially progressing to malignancy.^102^ The aetiology of the respiratory disease is less clear, with the simplistic interpretation that AAT deficiency contributes via loss of its capacity to neutralise neutrophil elastase. However, the lung phenotype of AAT individuals with complete serum deficiency varies markedly, and there is evidence for respiratory epithelial cell ER stress in the human disease and in mice transgenic for the ZZ‐AAT genotype. The human Z allele is a deletion which results in an amino acid substitution changing the conformation of the AAT molecule, the ZZ genotype is responsible for 98% of AAT represents the most severe emphysema.^103^ Furthermore, in cultured AAT mutant epithelial cells, the ER stress and consequent release of inflammatory cytokines and chemokines are exacerbated by exposure to cigarette smoke, consistent with the enhanced disease in smokers.^103^

## Oxidative and ER stress in acute respiratory conditions

Oxidative stress and ER stress have been implicated in the pathophysiology of a variety of more acute conditions affecting the lung. In septic shock and lung stress induced in animal models by exposure to LPS and other TLR ligands, both oxidative stress and ER stress occur in the damaged lung and appear to be a feature of the pathological process.^104^, ^105^, ^106^ In sepsis/LPS‐induced lung injury, both ER stress and autophagy (which may be a response to ER stress) occur and the systemic factor cold‐induced RNA‐binding protein (CIRP), local inflammatory cytokines such as IL‐17 and neutrophil activation have been linked with the development of ER stress and activation of the UPR.^104^, ^105^, ^106^ Pulmonary arterial hypertension results in chronic hypoxia, inflammation, and consequently oxidative and ER stress, and induction of autophagy, in the respiratory mucosa.^107^ In contrast, bronchopulmonary dysplasia (BPD) is a condition affecting ventilated premature neonates that develops as a result of hyperoxia and also appears to involve ER stress. In animal models of BPD hyperoxia in neonates drives ROS production, ER stress and ER stress‐induced apoptosis in the alveolar epithelium providing an explanation for the alveolar damage characteristic of BPD.^108^, ^109^

## Therapeutic approaches to resolve oxidative and ER stress in respiratory disease

The development of oxidative and ER stress in lung diseases described above suggests that therapeutic approaches to either dampen oxidative and ER stress, or modify the UPR, may be successful. Anti‐oxidants have relieved ER stress and pathology in some animal models of respiratory disease. For example, the strong anti‐oxidant, chlorogenic acid, suppressed ER stress, apoptosis and fibrosis in bleomycin‐induced fibrosis in mice.^86^ However, targeting oxidative stress in humans has been largely disappointing with the most evidence coming from trials of both airway and systemically delivered *N*‐acetyl‐cysteine (NAC) in CF, NCFBE and COPD.^110^, ^111^ Complicating matters, the limited beneficial effects seen including reduced exacerbation frequency and improved airway function are difficult to interpret as NAC has mucolytic as well as anti‐oxidant properties, and the beneficial effects may be attributed to improved mucociliary clearance rather than resolution of oxidative stress. Other approaches trialled include NOX‐inhibitors, superoxide dismutase‐mimetics, myeloperoxidase inhibitors and NRF2 activators; however, many of these drugs have failed in clinical trials and their development has been discontinued.^112^ New approaches or improved agents are required to suppress oxidative stress. For example, we have found that the cytokine, IL‐22, drives a robust natural anti‐oxidant programme in secretory cells,^113^ including respiratory epithelial cells, potentially providing a more effective mechanism to protect cells from both environmental and intrinsically‐generated ROS/RNS.

There have been reasonably intense efforts to therapeutically manipulate ER stress and the UPR for a broad range of diseases, including primary misfolding diseases, chronic inflammatory diseases, diabetes and cancer. There are several different classes of therapeutics including (1) modulators of specific arms of the UPR, (2) chaperone modulators, (3) chemical chaperones and (4) modulators of ERAD (for a review, see Rivas *et al*.^5^). Modulation of the UPR needs to be carefully considered as it could have complex consequences. For example, suppression of an arm of the UPR could reduce downstream inflammatory signalling and protect from apoptosis, providing benefit, but at the same time may impair the production of ER molecules involved in folding thereby exacerbating misfolding and driving the other arms of the UPR with potentially adverse consequences. Use of these experimental drugs in respiratory diseases is mainly restricted to preclinical models of disease and some examples are provided below.

Salubrinal is a selective inhibitor of the phosphatase‐mediated dephosphorylation of eIF2α which is downstream of PERK and is responsible for repression of translation during ER stress. Salubrinal has been demonstrated to repress cigarette smoke‐induced airway epithelial ER stress which is relevant for COPD therapy.^98^ 4‐phenylbutyrate (4‐PBA) and tauroursodeoxycholic acid (TUDCA) are chemical chaperones which promote correct folding in the ER that have been used widely in animal models of ER stress. 4‐PBA reduced ER stress, inflammation and fibrosis in the bleomycin model in mice,^84^ attenuated symptoms of asthma in the house dust mite allergen model in mice^90^ and also reduced ER stress and autophagy in LPS‐induced acute lung injury.^104^ Similarly, TUDCA has also been demonstrated to alleviate ER stress and associated pathology in the bleomycin fibrosis model and in asthma models in mice.^83^, ^92^ Whilst these studies provide proof of principle that targeting ER stress may be beneficial in human disease, we await the development of more specific, less toxic and more effective drugs, followed by clinical trials in the range of respiratory conditions that may benefit from these agents.

## Summary and conclusions

Oxidative stress and ER stress are frequent and clearly linked phenomena in a wide range of acute and chronic respiratory conditions, and there is substantial evidence that they are integral components of the pathophysiology of these conditions. More often than not oxidative stress and ER stress are accompanied by inflammation, both because the stress can be a consequence of factors produced by activated leucocytes, and because the cellular pathways activated by oxidative or ER stress lead to the release of factors driving both innate immunity and adaptive immunity. This potentially sets up a forward‐feeding cycle of cellular stress and inflammation, and in each specific respiratory condition, there is a need for a deeper understanding of the relative primary importance of inflammation and oxidative/ER stress, and whether targeting individual or a combination of these pathways will best reverse the pathological processes. Such studies are hampered by the lack of sophisticated therapeutic agents to modify oxidative and ER stress, with refinement of the current agents and approaches required before benefits can be realised in the clinic.
